# Impact investing market on Twitter: influential users and communities

**DOI:** 10.1007/s41109-018-0097-9

**Published:** 2018-09-26

**Authors:** Petra Kralj Novak, Luisa De Amicis, Igor Mozetič

**Affiliations:** 1 Department of Knowledge Technologies, Jožef Stefan Institute, Jamova 39, Ljubljana, Slovenia; 2 PlusValue, 131–151 Great Titchfield Street, London W1W 5BB, United Kingdom

**Keywords:** Impact investing, Sustainable investment, Social innovation, Social network analysis, Retweet networks, Community detection, Social influence

## Abstract

The 2008 financial crisis unveiled the intrinsic failures of the financial system as we know it. As a consequence, impact investing started to receive increasing attention, as evidenced by the high market growth rates. The goal of impact investment is to generate social and environmental impact alongside a financial return. In this paper we identify the main players in the sector and how they interact and communicate with each other. We use Twitter as a proxy of the impact investing market, and analyze relevant tweets posted over a period of ten months. We apply network, contents and sentiment analysis on the acquired dataset.

Our study shows that Twitter users exhibit favourable leaning (predominantly neutral or positive) towards impact investing. Retweet communities are decentralised and include users from a variety of sectors. Despite some basic common vocabulary used by all retweet communities identified, the vocabulary and the topics discussed by each community vary largely. We note that an additional effort should be made in raising awareness about the sector, especially by policymakers and media outlets. The role of investors and the academia is also discussed, as well as the emergence of hybrid business models within the sector and its connections to the tech industry. This paper extends our previous study, one of the first analyses of Twitter activities in the impact investing market.

## Introduction

Since 2000, a new international movement, led by public institutions and private capital along, has started emerging and growing. The objective was to have a financial system that could better serve society and tackle societal challenges, such as climate change, mass migration and aging populations.

This innovative wave continued to grow until 2007, when the term “impact investing” was coined and since then, an increasing number of policy makers, traditional investors, investees and not-for-profit organisations have shown interest, curiosity, passion or skepticism towards it. Many have started to talk about a proper “impact investing market”, despite the lack of a universal definition or agreed impact measurement methodologies.

For the purpose of this paper, the Global Impact Investing Network (GIIN) definition (GIIN 2018) will be used: “Impact investing consists of investments made into companies, organisations, and funds with the intention to generate social and environmental impact alongside a financial return”. Impact investment seeks below-market or market-rate returns and it differs from grants which are simply donations of funds with no expectation of financial returns.

**Fig. 1:**
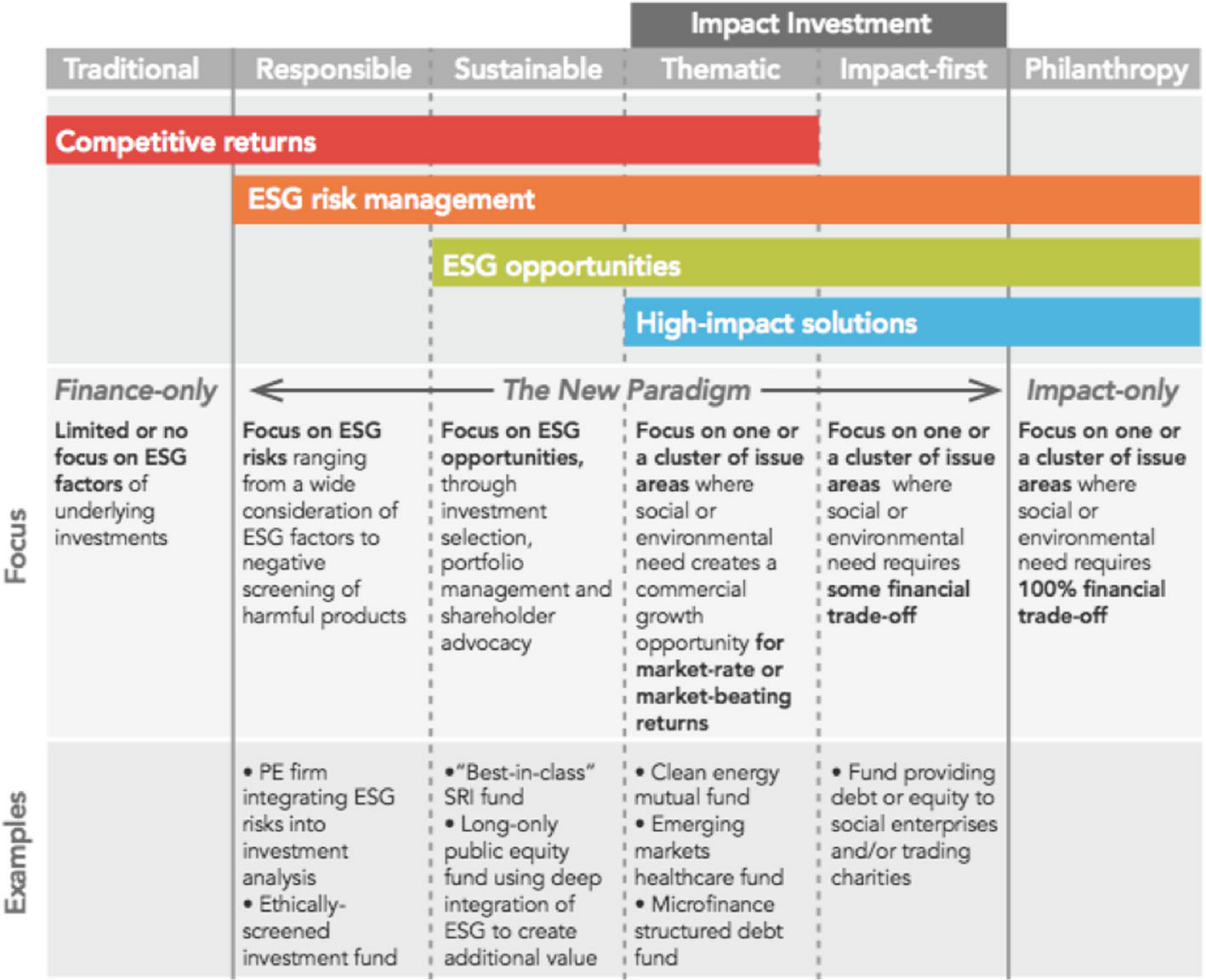
The impact investment spectrum. The investment spectrum ranging from traditional finance-only investments on the left hand side to impact-only philanthropy on the right hand side. Impact investing represents the new ‘middle-ground paradigm’ comprising of different sub-divisions (impact-first, thematic, sustainable and responsible investment)

In the “investment spectrum”, ranging from traditional finance-only investments to impact-only philanthropy, impact investing represents the new ‘middle-ground paradigm’ comprising of different sub-divisions (impact-first, thematic, sustainable and responsible investment), as shown in Fig. 1 (Ventures 2015).

While still representing a relatively small portion of total investments, the fast growth rate of the sector is impressive. According to the GIIN, the 2016’s total impact investment (114 billion US$ reported by 208 investors) registered an increase of 48% from 2015 (77 billion US$ in total assets reported by 158 investors) and 90% from 2014 (60 billion US$ in assets reported by 146 investors). According to Bloomberg Brief, socially responsible investments worldwide grew by 25% to 23 trillion US$ over the last two years, with particularly strong growth in China, Japan, Australia and New Zealand. As for Europe, Eurosif — the pan-European sustainable and responsible investment membership organisation — reports that impact investing is growing at a compound annual rate of 120%. In 2015, 98 billion Euros were invested, up from only 20 billion Euros in 2013, and forecasts remain positive.

Since the impact investing market is currently growing, there is a strong incentive and a widely recognized need of making it as interconnected and functional as possible. Within this scope, the goal of this study is to collect and analyse contributions from Twitter users that are operating in the impact investing market to ultimately provide evidence-based recommendations for fostering the existing ecosystem and raising awareness around the topic. We use Twitter as a proxy of the impact investing market and apply complex networks analyses and sentiment analysis.

More specifically, we address the following research questions:

- Does, and to what extend, impact investing attract social media attention?
- Who are the key influencers and their communities? What are the most important topics that they discuss?
- What are the relationships and interactions between different categories of players? Consequently, how cohesive is the investing market?

A preliminary study of the same topic was already published at a conference (Kralj Novak et al. 2017). At the best of authors’ knowledge, this is the only attempt that has been made to try to understand the market’s players and interactions by using big data. The extensions, presented here, in comparison to the conference paper, include:

- considerably larger dataset (time period extended from 106 days to 306 days, Twitter volume increased from 234,243 to 668,529 tweets);
- extended annotated dataset (manual categorisations of 520 influential users);
- analysis of the community structure in the retweet network;
- analysis of the community bonding vs. community bridging links.

As a methodological novelty, we adapt the Herfindahl-Hirschman index (*HHI*) to the retweet communities to detect the community structure. *HHI* is a measure of market concentration commonly used in economics to measure the amount of competition among leading companies in an industry with respect to their market share (Werden 1998). We combine the *HHI* with the Twitter user influence as measured by the adapted Hirsch index (*h-index* (Grčar et al. 2017)) to study the structure of the communities. The results show that *HHI* can be used to quantify how hierarchical a community is.

This paper is organized as follows. In “Twitter volume and sentiment” section, the data acquisition process and the collected Twitter data are presented. “Twitter users: influence and categories” section discusses the notion of influencers on Twitter and identifies the main influencers in the impact investing market. “Retweet communities” section discusses the communities in the retweet network, their characteristics and diversity. “Content analysis” section investigates the content of the tweets in terms of the hashtags used. “Discussion” section discusses the findings, provides insights gained, and suggestions of improving the communication between the key players in the impact investing market. We conclude in “Conclusions” section.

## Twitter volume and sentiment

We used the Twitter Search API to acquire tweets. In ten months, between March 28, 2017 and January 28, 2018 (306 days) we collected 668,529 tweets. The number of distinct Twitter users in the dataset is 134,482. The search query is a combination of a list of well-known individuals and organisations working in the impact investment sector, and a list of relevant Twitter hashtags. These were used to seize and explore the Twitter coverage of impact investing.

In particular, the queries include relevant users (e.g., @YF_Academy, @esmeefairbairn, @resonanceltd, @Big PotentialSI, etc.), single hashtags (#socfin, #impinv #socialfinance, #impactinvestment, etc.), combined hashtags (#social & #finance, #social & #investment, #impact & #assessment, etc.), and hashtags of major impact investing events (#impact2, #socap17, #OxfordIIP, #skollwf, etc.).

**Fig. 2:**
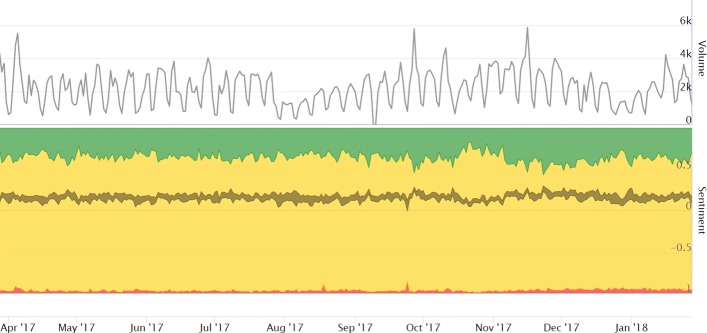
Volume and sentiment of collected tweets. The top chart shows the daily volume of the tweets acquired, while the bottom chart shows the aggregated sentiment of tweets. Sentiment is computed from a general purpose English sentiment model: positive sentiment is in green, neutral sentiment in yellow, negative sentiment in red, and the mean sentiment score is in gray

The upper chart of Fig. 2 shows the volume of tweets. On average, there are about 2,200 tweets per day. A weekly seasonality can be observed with high volumes on working days and low volumes on weekends. The daily number of tweets is affected by summer holidays in the northern hemisphere and the Christmas/New Year holiday season as well. The two highest volume peaks were on November 16, 2017 (5864 tweets), during the Social Entreprise Day, and on September 27, 2017 at the launch of the Nasscom Social Innovation Forum (5789 tweets). Overall, 2200 tweets per day is a low volume in comparison to more popular topics. For example, environment related tweets show stable volume of about 200,000 posts per day (Sluban et al. 2015), while interesting events, like Brexit, are trending for a short time, with more then one million tweets on the referendum day (Grčar et al. 2017). The volume of the tweets related to impact investing shows both patters, stable and trending, but the overall volume is low since this is not a mainstream topic.

We applied a domain independent sentiment classification model to the corpus of English tweets, to obtain an overview of the sentiment polarity and subjectivity of Twitter users regarding impact investing. Our approach to automatic sentiment classification is based on supervised machine learning. The procedure consists of the following steps: (i) a large sample of tweets is first manually annotated with sentiment by humans, (ii) the labeled set is used to train and tune a classifier, (iii) the classifier is evaluated by cross-validation and compared to the inter-annotator agreement, and (iv) the classifier is applied to the whole set of collected tweets. In particular, we developed a supervised machine learning classifier based on a linear kernel SVM (Mozetič et al. 2016). The classifier was trained on 90,000 manually labeled English tweets. The tweets are first processed into the standard Bag-of-Words (BoW) representation, which includes advanced tokenization to correctly handle emoticons, emojis, urls and mentions, lemmatization, frequent unigrams and bigrams, TF-IDF weighting, stop-word removal, and normalization of vectors. The classifier, named TwoPlaneSVMbin, consists of two SVM classifiers: One classifier is trained to separate the negative tweets from the neutral-or-positives; the other separates the negative-or-neutrals from the positives. During classification, the distances from both hyperplanes determine the appropriate bin, and the class is determined by the majority of the training instances in the bin.

In our previous study (Mozetič et al. 2016) we compared five variants of the SVM classifiers and Naive Bayes on the Twitter sentiment classification task. TwoPlaneSVMbin was always between the top, but statistically indistinguishable, best performing classifiers. It turns out that the quality of the annotation process has much larger impact on the performance than the type of the classifier used. In particular, annotations have to be language- and domain-specific. For example, Qi et al. (2018) show an application of a Chinese-specific sentiment classifier to investor sentiment about energy stocks. In Ranco et al. (2015); Gabrovšek et al. (2017) we develop a classifier specific for US stock trading vocabulary and apply it to 30 Dow Jones companies. However, domain-specific annotations require considerable resources, and in the current paper we apply a generic, domain independent sentiment classifier for English.

The sentiment classification results are provided at the bottom of the chart in Fig. 2, showing aggregated sentiment scores of tweets per day. While the majority of the tweets present neutral sentiment towards impact investing, the subjective tweets (non-neutral) are predominantly positive, and the mean sentiment score (grey line) is also positive. This suggests that most of the users tweeting about impact investing do not have or show strong sentiment about it, but those who are not neutral, tend to have a positive sentiment.

The fact that most of the tweets are neutral can be explained by the fact that they mostly occur during the workdays and that they are mainly aimed at sharing information and pointing to relevant resources. However, the general positivity of the non-neutral tweets might come as a surprise. Namely, a previous study of sentiment analysis of environmental topics (Sluban et al. 2015), shows that different communities have very diverse range of sentiment leanings towards climate change on one hand, and oil, gas and fracking on the other hand.

## Twitter users: influence and categories

In this paper we treat retweeting as a key action for the diffusion of information on Twitter and the establishment of influence trends. We analyze two distinct aspects of retweeting activities. In this section, we track the social influence of Twitter users by looking at their posting activity and their ability to attract attention through follower engagement (i.e., rate of the posts retweeted). In the next section, we construct a retweet network where Twitter users are linked if they retweet each other. Building on that, we can identify the most prominent communities in the network and pinpoint the role played by the influential users within them.

A retweet is a form of interaction between Twitter users, when a user re-posts an already posted tweet, with an attribution to the original tweet. In a retweet of a retweet (or any further retweets), the attribution is always given to the original, thus eliminating all intermediate retweeting actions. When a user retweets a tweet, it is distributed to all the followers, just as if it were an originally authored tweet.

There are three main modalities in which users on Twitter interact: (i) the user follows posts of other users, (ii) the user responds to other user’s tweets by mentioning them or replying to them, and (iii) the user forwards interesting tweets by retweeting them. Based on these three interaction types, one can define three measures of influence of a Twitter user (Cha et al. 2010): *indegree influence* (the number of followers, indicating the size of the audience), *mention influence* (the number of mentions of the user, indicating the ability to engage others in conversation), and *retweet influence* (the number of retweets, indicating the ability of the user to write content of interest to others).

Kwak et al. (2010) compare three different network-based measures of influence on Twitter: the number of followers, PageRank, and the number of retweets. They find that the ranking of the most influential users differ considerably depending on the measure. Cha et al. (2010) also compare three different measures of influence: the number of followers, the number of retweets, and the number of mentions. They also find that the most followed users do not necessarily score the highest on the other measures. Wang et al. (2010) compare the number of followers and PageRank with a modified PageRank measure that accounts for topic, again finding that ranking depends on the influence measure. Suh et al. (2010) investigate how different factors, such as the account age, the use of hashtags and URLs impact the influence of the user measured by the number of retweets. Bakshy et al. (2011) investigate how information spreads on a retweet network and whether there are preconditions for the user to become influential. Boyd et al. (2010) examine retweets as a conversational practice and note that retweeting can be understood both as a form of information diffusion and as a means of participating in a conversation.

The related work indicates that retweeting most closely reflects the intuitive notion of engaging others and getting support on Twitter. However, the retweet influence alone ignores the productivity of the Twitter user. Therefore, we combine the ability of the user to produce original contents about relevant topics with the contents spreading in the form of retweet influence. This combination resembles the scientific influence, therefore we adapt the well-known Hirsch index to measure the social influence on Twitter (Grčar et al. 2017).

The Hirsch index (*h-index*) (Hirsch 2005) is here adapted and used in order to rank relevant Twitter users by their social influence in the network. The *h-index* is a well-known author-level bibliometric indicator that measures the scientific output of a scholar by quantifying both the number of publications (i.e., productivity) and the number of citations per publication (i.e., citation impact). We apply the *h-index* to our dataset of Twitter users (Grčar et al. 2017): a user with an index of *h* has posted *h* tweets and each of them was retweeted at least *h* times. Let *RT* be the function indicating the number of retweets for each original tweet. The values of *RT* are ordered in decreasing order, from the largest to the lowest, while *i* indicates the ranking position in the ordered list. The *h-index* is then defined as follows:

$$h-index(RT) = \max_{i} \min(RT(i), i). $$

A survey of influence measures on Twitter is provided in Riquelme and González-Cantergiani (2016). Twitter *h-index* was already used to measure the influence of proponents and opponents of Brexit (Grčar et al. 2017). In King et al. (2013) it was referred as T-index.

The top ten most influential Twitter users (with *h-index*≥14), are in Table 1. Each user is also assigned to one of the categories which designates different types of actors in impact investing. These categories were devised manually by impact investing experts (see Table 2).

**Table 1:** Top ten influential Twitter users (by *h-index*) along with their user category and description

Twitter user	*h-index*	Category	Description
@jalloyd4	69	Practitioner	John Lloyd IV, CMO of @ClearlySo, Board Member of @eCadets
@ClearlySo	66	Intermediary	Raises capital, runs impact investing network
@IgnacioMls	23	Practitioner	Ignacio Mesalles: Solving environmental and social issues
@SchSocEnt	20	SocialBusiness	School for Social Entrepreneurs, charity that supports entrepreneurial approaches
@atlcelebrity	19	Practitioner	Dr. Dionne Mahaffey: CEO @TalkGoodBiz @WhereUCameFrom |AKA1908 Entrepreneur, PsyD, Volunteer, Techie | Forbes Coaches
@SocialEnt_UK	19	Intermediary	Social Enterprise UK is the membership body for social enterprise
@GoldmanSachs	17	Investor	Official Goldman Sachs Twitter account
@helene wpli	17	Practitioner	Helene Li: Doing good while doing well... #socialchange catalyst, #strategy #entrepreneurship consultant, #banking #UHNW #NextGen
@SocEntGlobal	14	Intermediary	The British Council’s programme to link UK social enterprise with the global sector, in policy and practice.
@2morrowknight	14	Practitioner	Sean Gardner: World Traveler, VP of Digital @WorldCommForum, Board Member @DigiMarketingWF, Keynote Speaker, #GivingTuesday Ambassador; shooting a film with producer @RashaGoel

**Table 2:** Twitter user categories, the number of influential users (with *h-index*≥5) in each category, and its description

Category	No. of influential Twitter users	Description
Investor	40 (8%)	Banks, funds, asset managers
Social business	69 (13%)	Any organisations or enterprises (for- profit or not-for-profit) with a social purpose, such as NGOs, charities, voluntary and community organisations, social enterprises, community interest companies (CIC)
Practitioner	141 (27%)	Individuals working independently or for organisations active in the field, opinion leaders
Ad-hoc initiative	25 (5%)	Activities relevant for the sector with no legal status, such as one-off funded projects, policy initiatives, steering groups
Intermediary	151 (29%)	Any organizations that do not tackle a social problem directly but enable other players to do that, such as Foundations, fairs, business support hubs, national membership bodies, platforms, networks
Private company	29 (6%)	For profit private companies excluding social enterprises
Media	29 (6%)	Magazines, blog, podcasts, journalists
Public sector	13 (3%)	Local or national government, international institution
Academia	14 (3%)	Universities, professors, researchers
Other	9 (2%)	Individuals or organisations doing primarily something unrelated to the topic, political parties
Total	520 (100%)	

As for the most influential Twitter users, John Lloyd IV (the Chief Marketing Officer of Clearly So) and Clearly So itself, have the highest *h-index* (respectively 69 and 66). The third most influential account is from Costa Rica (Ignacio Mesalles) and the one on the eighth position is from Hong Kong/Singapore. All the others in the top ten are either from the UK, more specifically from London (five), or US (three). This can be partially explained by the fact that all the Twitter queries were in English, but also by the fact that the UK and US share a leading position at the global level in the impact investing field. Half of the top ten Twitter accounts are practitioners, whilst the other half are organizations, out of which three are intermediaries, one is a social business and one is an investor. Consistent with the previous study (Kralj Novak et al. 2017), no journalists, media outlets, academia nor the public sector are in the top ten positions, despite their vested interests. Goldman Sachs is the seventh most influential account, confirming that many “traditional investors” are moving towards or showing a growing interest in impact investing. However, to find an influential traditional investor in such a prominent position comes as a surprise, especially considering the natural aversion of this kind of organizations towards social media.

There is only one social business among the most influential Twitter users, namely the School for Social Entrepreneurs. The school supports social entrepreneurs, intrapreneurs and charity leaders and runs courses that equip them with the skills and networks needed to create lasting change. Foundations are surprisingly missing in our top league. Finally, the tenth account is from a Twitter user Sean Gardner, who defines himself as “world traveller, speaker, film shooter”, and ultimately a communicator. Yet, he is also an Ambassador for the #GivingTuesday initiative, and, with his 927,514 followers, he is an excellent promoter for the sector, precisely because of his “outsider” position.

Further analyses were done on all the 520 Twitter users with *h-index*≥5. Firstly, the 520 users were manually categorised according to the nature of the activities they run. All the categories are listed in Table 2, showing Twitter user categories, the split across categories (in absolute and relative terms), and a description (including examples) of each category.

- *Investor* (8% of the users): If we consider the absolute number, we see that impact investing topics have been mentioned by 40 investors with influential Twitter accounts across the world, from Europe to the US and Canada. The range includes very specialized funds and big mainstream investment banking institutions. The concept is spreading, yet the percentage represented by the entire sample is still relatively low. This can be explained either by the nature of the investor-investee relationship (few to many) or by the fact that Twitter is often not the investors’ preferred channel of communication.
- *Social Business* (13%): Social businesses are increasingly using social media to promote their activities and thus as a vehicle to raise public awareness. Most of our users in this category are social enterprises aiming to deliver services that can significantly vary, from renewable energy production to education for tackling unemployment, through new business models such as a socially inclusive letting agency or an environmental-friendly bus service.
- *Practitioners* (27%): This is the second largest group and is mainly represented by individual professionals in the sector (such as CEOs, founders, senior managers) who are active on Twitter in their personal capacity.
- *Intermediaries* (29%): As expected, this is the largest category identified within the sample. It includes all the so-called “enablers”, which due to their very nature and intrinsic purposes aim to communicate relevant information for the sector, whilst raising awareness with the broader public. This does not mean to indicate that (online) communication is their only objective, as the diversity within this group is broad, ranging from foundations to membership bodies, from India to Nigeria, through Australia and UK, but it is something common across all the user group.
- *Private Company* (6%): With regards to this relatively small user group, it should be remarked that 27% of the sample has a focus on tech. They are either companies that use new technologies to collect and analyse impact-related data (i.e., TruValueLabs), or they are “traditional” IT-based companies (i.e., Atos) that pay significant attention to impact. This group also includes consultancy companies (i.e., KPMG and Capgemini) that are working on projects related to impact investment.
- *Media* (6%): This category includes journalists, magazines and contributors writing about the topic. Notably, the dataset not only includes users such as the representative from Bloomberg Brief (as in our previous study), but also Twitter accounts such as the BBC World or Forbes India. Yet, there are not so many mainstream media as expected and sector-specific media still play the largest role.
- *Public sector* (3%): In line with the previous study, users from the public sector tweeting about the topic accounts for a low percentage. However, what is surprising is that 7 out of the 13 Twitter accounts are from the EU institutions and 3 are from the UN agencies and programmes. Only three belong to national governments (Canada, India, US), and the UK is missing from the statistics, despite being considered one of the leaders in the sector at the global level (and the dataset containing English tweets only).
- *Academia* (3%): The sample includes both individual professors and Twitter accounts for different university courses. Although many good universities nowadays have programmes and classes on impact investing, social entrepreneurship or social innovation, not so many of them are active on social media to promote or share info about their courses online. All of those in the sample are from the UK and US, except two from Germany.

The presence of user categories like *Ad-hoc Initiatives* or *Others* shows that the sector is receiving attention from users outside the impact investing sector itself, but that they do not yet exhibit a coherently structured approach.

## Retweet communities

In this section we show how the collected tweets are used to construct a retweet network. A retweet network is a directed weighted graph, where nodes represent Twitter users and edges represent the retweet relations. The direction of an edge corresponds to the direction of information spreading or influence. The weight of an edge is the number of times one user retweets the other.

The directed weighted graph is used to identify the most influential users, in terms of *h-index* (as in the previous Section), where the number of retweets is important. When we collapse the weighted edges into unweighted, we consider just relations between the Twitter users, who retweets whom. A further abstraction ignores the direction of the edge: two users are linked if one retweets the other one, but the source and destination are irrelevant. Note that for the retweet relation, there is an edge between the author of a tweet (source) and the user who retweeted it (destination) even indirectly, thus ignoring all intermediate retweets. It turns out that such undirected, retweet graph between the Twitter users is useful to detect communities of like-minded users who typically share common views on specific topics.

In complex networks, a community is defined as a subset of nodes that are more densely linked between themselves than with the other nodes. Several definitions of communities and different methods to identify them were proposed, see Fortunato (2010) for a review. A more user oriented review that also provides strengths and weaknesses of the most popular methods with directions for their use is provided in Fortunato and Hric (2016).

For the purpose of this paper, we apply a standard community detection algorithm to our undirected retweet network, the Louvain method (Blondel et al. 2008). The method partitiones the network nodes into communities with the goal to maximize its modularity. The modularity measures the community density and structure within the network: the fraction of edges within groups of a given network partitioning, as compared to the expected fraction of edges in the groups if edges were randomly distributed in the network (Newman 2006). The Louvain method is a computationally very efficient algorithm, well suited for large networks, and allows (i) to analyze large networks with good scalability and (ii) to avoid ex-ante assumptions about their size or the number of communities (Lancichinetti and Fortunato 2009). We have already successfully applied the Louvain method for community detection to uncover influential communities in retweet networks in the context of climate and energy issues (Sluban et al. 2015). Our application and evaluation of the Louvain method on a retweet network of the European Parliament shows that there is a high degree of match between the detected retweet communities and the political group membership and nationalities of the members of the Parliament (Cherepnalkoski and Mozetič 2016).

**Fig. 3:**
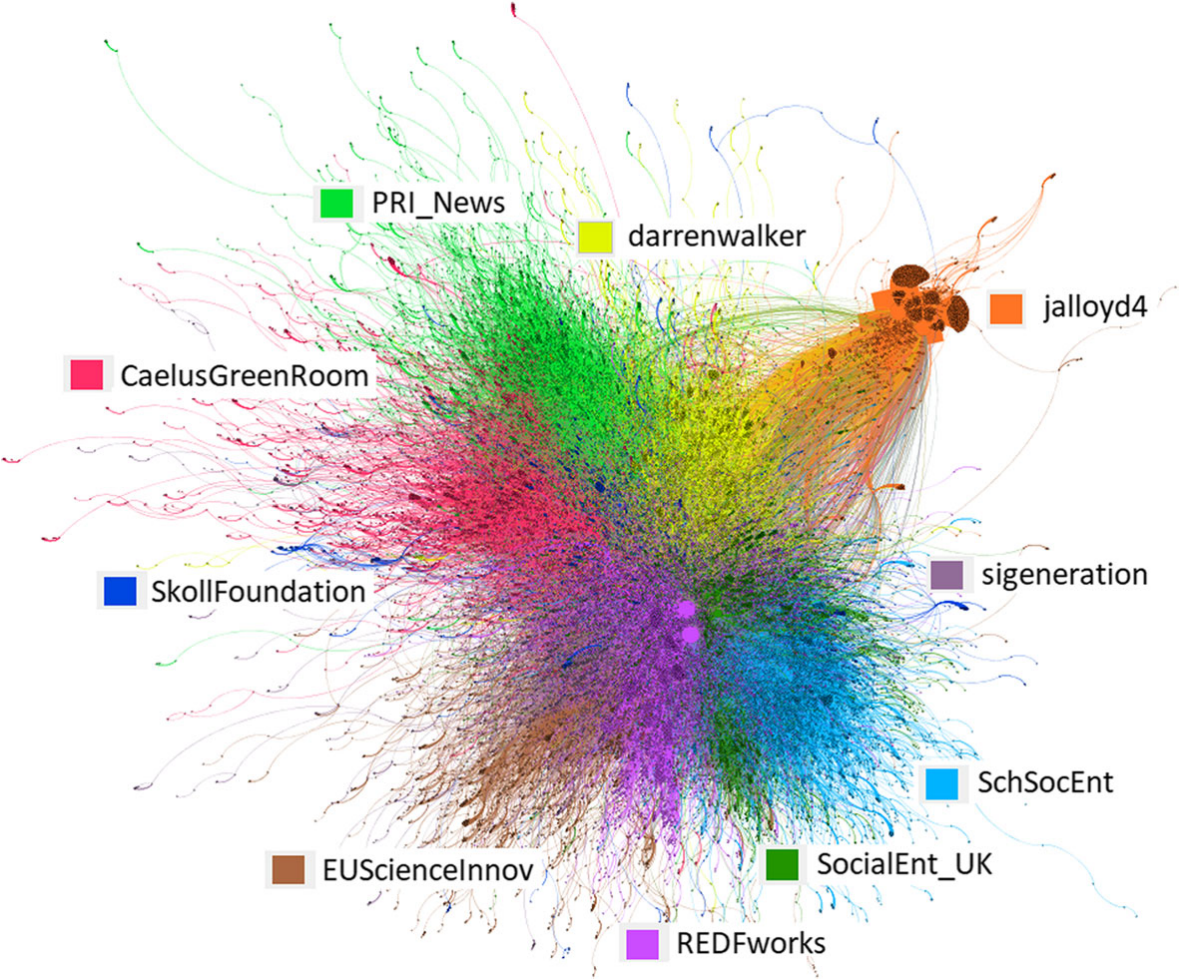
Retweet communities, each identified with the most influential Twitter user, as measured by the *h-index*

From the collected tweets on impact investing, we construct a retweet network, comprising of 120,858 nodes (Twitter users) and 210,337 directed edges (retweets). When directed edges are merged into undirected, the network contains 206,231 undirected edges, just 2% less. The giant connected component of the network has 107,908 nodes and 197,551 directed edges. The Louvain community detection (resolution=1) was run on the giant connected component and yielded ten communities with more than 3,000 nodes each (with modularity value 0.69, accounting for 58% of all the users and 65% of the users in the giant connected component). The detected communities are presented in Fig. 3.

The detected communities are often led by the most influential Twitter users, as identified in Table 1. Properties of the most influential users and the corresponding retweet communities are in Table 3. We observe the following:

**Table 3:** Properties of most influential (central) users (left-hand side), and the corresponding retweet communities (right-hand side)

id	Central users	*h-index*	Retweeted by no. of users	Retweeted by no. of times	No. of nodes (comm. size)	No. of influential users (*h-index*≥5)
0	@SchSocEnt	20	1125	2588	6821	50
2	@jalloyd4	69	6859	11573	10914	7
4	@darrenwalker	13	417	498	6855	47
8	@SkollFoundation	13	674	978	3494	24
9	@REDFworks	6	160	313	12419	14
10	@PRI_News	11	334	576	6319	41
11	@CaelusGreenRoom	11	188	1041	6335	16
18	@SocialEnt_UK	19	1368	2416	6811	83
26	@sigeneration	9	170	292	3193	27
30	@EUScienceInnov	9	136	180	3579	20

- Three out of the ten communities include one of the ten most influential Twitter users of Table 1 (i.e., *h-index*≥14).
- Other four communities include one of the top 20 users (i.e., *h-index*≥11).
- Only one, albeit the largest, community is led by a user with a relatively low *h-index*=6, namely @REDFworks.

We examine the differences in the structure of the detected communities *C*_1_,…,*C*_*n*_ by measuring the distribution of influence among the community members. We use the Herfindahl–Hirschman index (*HHI*) to measure distribution of the user influence within a community. In economics, *HHI* is a measure of market concentration, commonly used to measure the amount of competition among leading companies in an industry with respect to their market share (Werden 1998). When applied in the context of community structure, we consider the *N* leading users *u*_*i*_, *i*∈{1,…,*N*}, in a community *C* in terms of their *h-index*:

$$HHI(C) = \sum\limits_{i = 1}^{N}{r_{i}^{2}} = \sum\limits_{i = 1}^{N}{\left(\frac{h-index(u_{i})}{{\sum\nolimits}_{j=1}^{N} h-index(u_{j})}\right)^{2}}. $$

The squared sum of influence ratios ranges from 1/*N* to 1, where lower values indicate a dispersed and more balanced influence distribution, whereas higher values reflect the community influence concentrated at only a few strongly influential users. Using the *h-index* in the computation of *HHI* is a novelty compared to previous work (Sluban et al. 2015) where the in-degree within the community was used as the user influence measure. We argue that the *h-index* is a better metric choice as it encompasses two dimensions: the author’s productivity (the number of original tweets posted) and the visibility (the number of retweets).

The *HHI* values for the analyzed communities are presented in Table 4, together with the number of unweighted edges within and outside the community (the number of users who retweeted) and weighted edges (the number of retweets). We note that one community (*id*=2) is very different from the rest. All the detected communities, except the one, have a very evenly distributed influence, with 0.1<*HHI*<0.125. Similar conclusion follows from the number of important users (*h-index*≥5) per community, where the count ranges from 7 in the most hierarchical community (*id*=2) to 83 in the community with the most distributed influence among its members (*id*=18). The communities are also very closed, with the ratio of edges outside vs. within the community around 0.05, both in terms of users (unweighted edges) and retweets (weighted edges).

**Table 4:** Structural properties of the main retweet communities. *HHI* is computed for *N*=10 top users in each community

			No. of unweighted edges			No. of weighted edges			
id	Central user	No. of nodes	within	outside	out/in	within	outside	out/in	*HHI*
0	@SchSocEnt	6821	16349	1913	0.117	32591	2578	0.079	0.123
2	@jalloyd4	10914	15747	412	0.026	27121	518	0.019	0.274
4	@darrenwalker	6855	16251	1034	0.063	24668	1240	0.050	0.103
8	@SkollFoundation	3494	6501	329	0.050	8793	366	0.041	0.112
9	@REDFworks	12419	23589	1709	0.072	35321	1951	0.055	0.100
10	@PRI_News	6319	12400	596	0.048	18356	689	0.037	0.103
11	@CaelusGreenRoom	6335	10514	698	0.066	15939	791	0.049	0.106
18	@SocialEnt_UK	6811	15980	1376	0.086	26540	1835	0.069	0.110
26	@sigeneration	3193	6873	245	0.035	9872	254	0.025	0.103
30	@EUScienceInnov	3579	5832	241	0.041	7830	264	0.033	0.102

The exceptional and very different community to the others in terms of the community structure is lead by John Lloyd (@jalloyd4). The community is star-shaped and concentrated around only seven influential users. Also its ratio of edges within and outside the community is only one half of the other communities. Out of the ten communities, only one is led by a media agent (@CaelusGreenRoom) or by an ad-hoc initiative (@PRI_News, the UN-supported Principles for Responsible Investment). All the others are led by practitioners or intermediaries.

## Content analysis

**Fig. 4:**
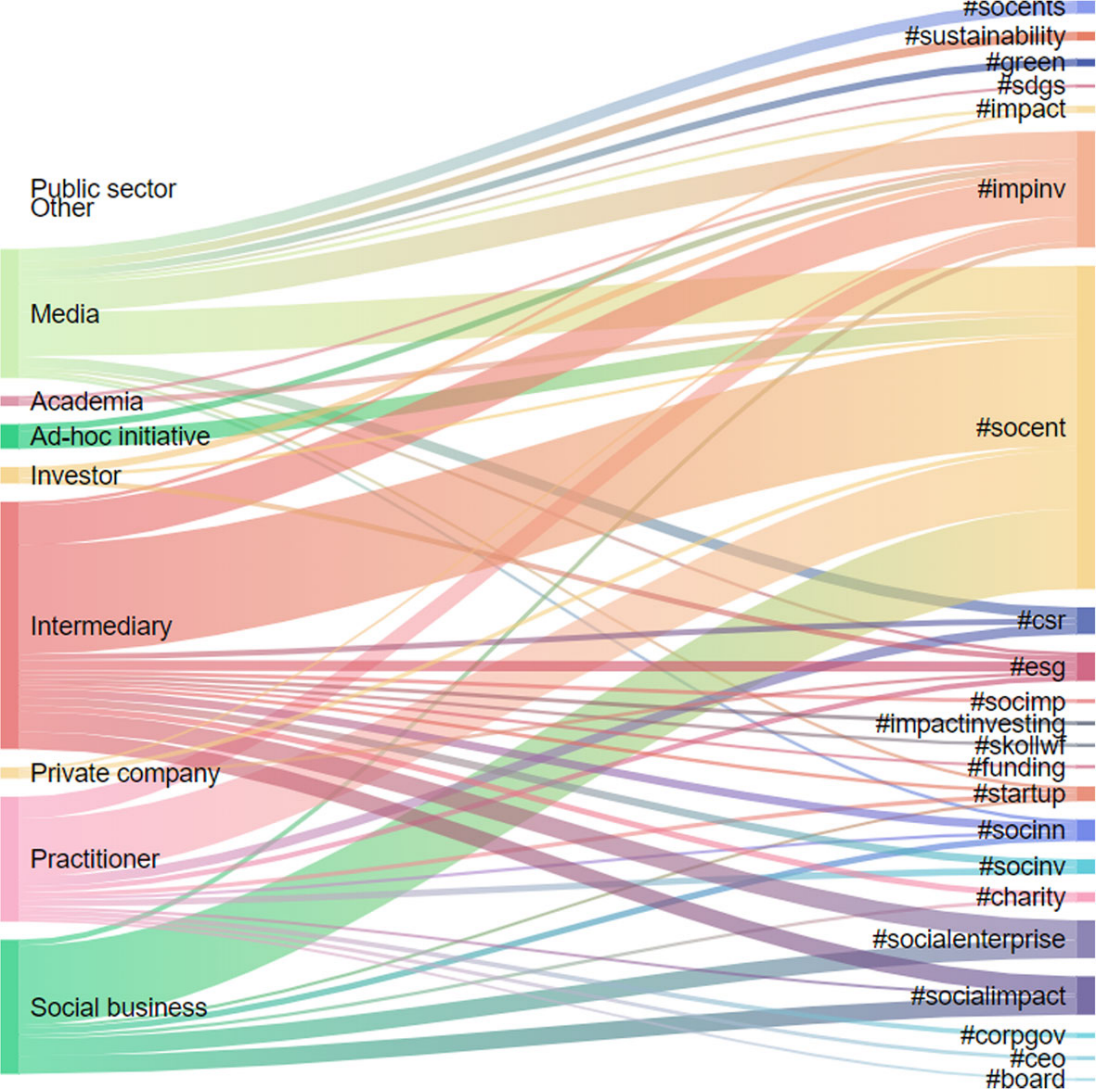
Mapping of the user categories (left-hand side) to hashtags (righ-hand side). The ten user categories comprise the top 520 influential users. Each hashtags is used at least 500 times

**Fig. 5:**
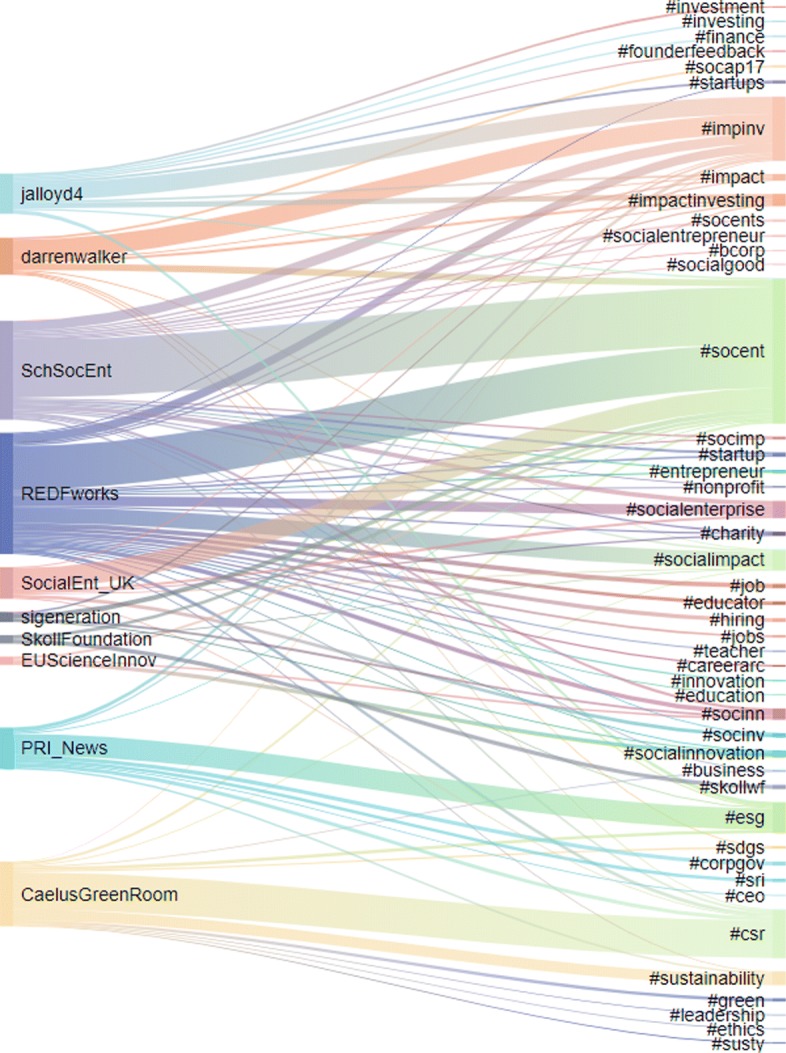
Mapping of the retweet communities (left-hand side) to hashtags (right-hand side). The retweet communities are identified by the most influential users. Shown are hashtags used at least 1000 times

**Fig. 6:**
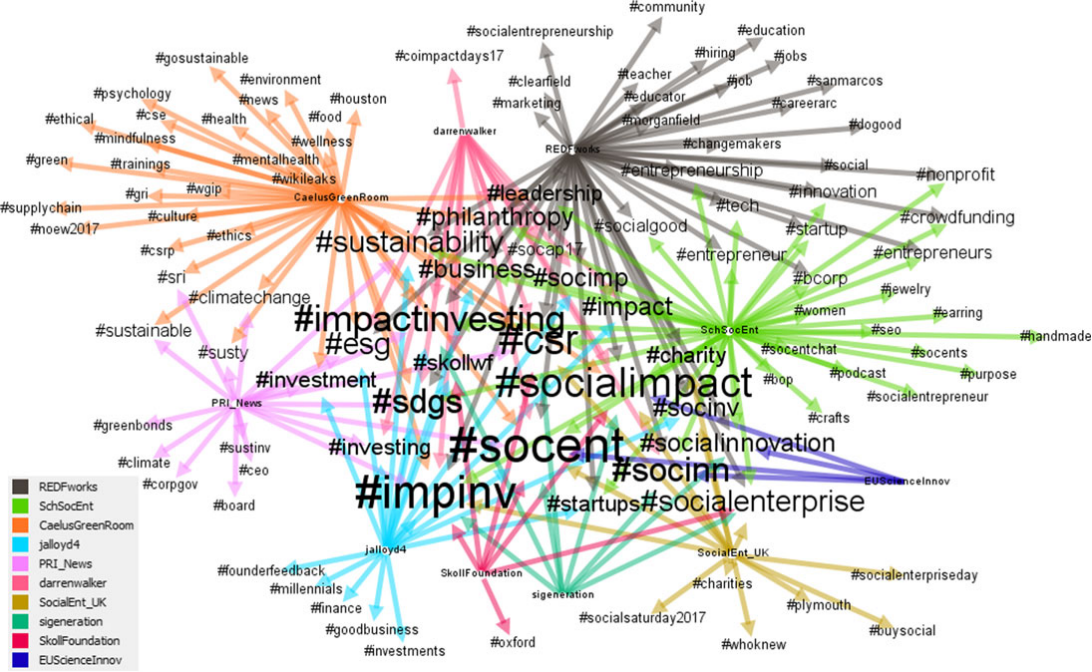
A bipartite network of the retweet communities and hashtags. The retweet communities are denoted by colors (bottom-right inset) and identified by the most influential users. Shown are hashtag used at least 500 times

In this section we present the content analysis of the user categories and the retweet communities in terms of their hashtag usage. We first look at the hashtags used by the 520 influential Twitter users grouped into the categories from Table 2. The resulting mapping is depicted in Fig. 4, with the threshold on the frequency of hashtags per category set to 500. We then look at the hashtags used by the users of different retweet communities at least 500 (Fig. 6) and 1000 (Fig. 5) times.

In Fig. 4, #socent (social enterprise) and #impinv (impact investing) are the most popular hashtags, tweeted by all eight user categories (who used any hashtag at least 500 times), followed by #esg (Environmental, Social and Governance) in 5 communities and #csr (Corporate Social Responsibility) in 3 communities. #startup is used by 4 user categories. #socialenterprise and #socialimpact are used mainly by Intermediaries and Social businesses. #sustainability, #green and #sdgs are frequently used by Media users only, despite the topics’ popularity. 11 out of the 22 hashtags in Fig. 4 have been tweeted by one category only. This can be interpreted as if different user categories of influencers tend to have or use a sectorial “Twitter vocabulary”. This does not necessarily signal that they represent “closed” categories, as they might belong to different communities — this will be further explored in the next section of the paper. Ad-hoc initiative, academia, private company and investor are the categories that have the least diversity in their Twitter vocabulary. The categories Public sector and Other did not use any hashtag more than 500 times.

The content in terms of hashtags used by the retweet communities is presented next. Figure 5 shows a Sankey diagram mapping of retweet communities and the most used hashtags. The width of the bars is proportional to the usage. Figure 6 shows a bipartite network of retweet communities and hashtags, with a lower threshold to allow for a more detailed analysis. The size of the hashtag is proportional to its usage.

In both figures, #socent (social enterprise) and #impinv (impact investing) are the most commonly used hashtags. #csr (Corporate Social Responsibility), #esg (Environmental, Social and Governance) and #socialenterprise also emerge from both figures but whilst #sdgs is only used by 2 communities in Fig. 5, the same hashtag, appear as central when we refine our analysis (Fig. 6). Among the hashtag used in both figures, there are also two important annual events for the sector #socap17 in San Francisco and #skollwf in Oxford.

Focusing more closely on Fig. 5, it emerges that #socent and #impinv are both used by all the retweet communities, whilst #csr, #esg and #socialenterprise are predominantly used in one community, respectively @CaelusGreenRoom, @PRI_News and @REDEworks and many of the hashtag are used by one or two communities only (32 out of 45). What can be drawn from Fig. 5 is that there is no common language across the communities and each community has its own “vocabulary”. #socent is the most popular expression across the user sample, followed by #impinv.

Figure 6 shows a bipartite network of the retweet communities within the network displaying hashtags that have been used at least 500 times. In comparison to Fig. 5 which displays hashtags used at least 1000 times, this figure allows us to give an overview of the topics discussed by the different retweet communities. It shows a greater diversity of hashtags used in terms of their environmental concerns (i.e., #climatechange, #sustainable, #environmental), education and job market (i.e., #teacher, #educator, #jobs, #hiring), or inspirational attitude/practice (#ethics, #mindfulness, #gosustainable). Furthermore, the figure also sheds light on the character and the potential targeted audience of the different communities within the network. For instance, the @SchSocEnt community seems to rely on hashtags that are related to enterpreneurs in the crafting business (#jewlery, #handmane, #enterpreneurs). Climate change emerges as a topic common to the @PRI_News and @CaelusGreenRoom communities. Education and job market, two key topics for the impact investing sector, appear only in the @REDFworks-led community, whilst impact assessment/measurement is totally missing in the figure — this is quite surprising considering how relevant the topic is for the sector.

**Fig. 7:**
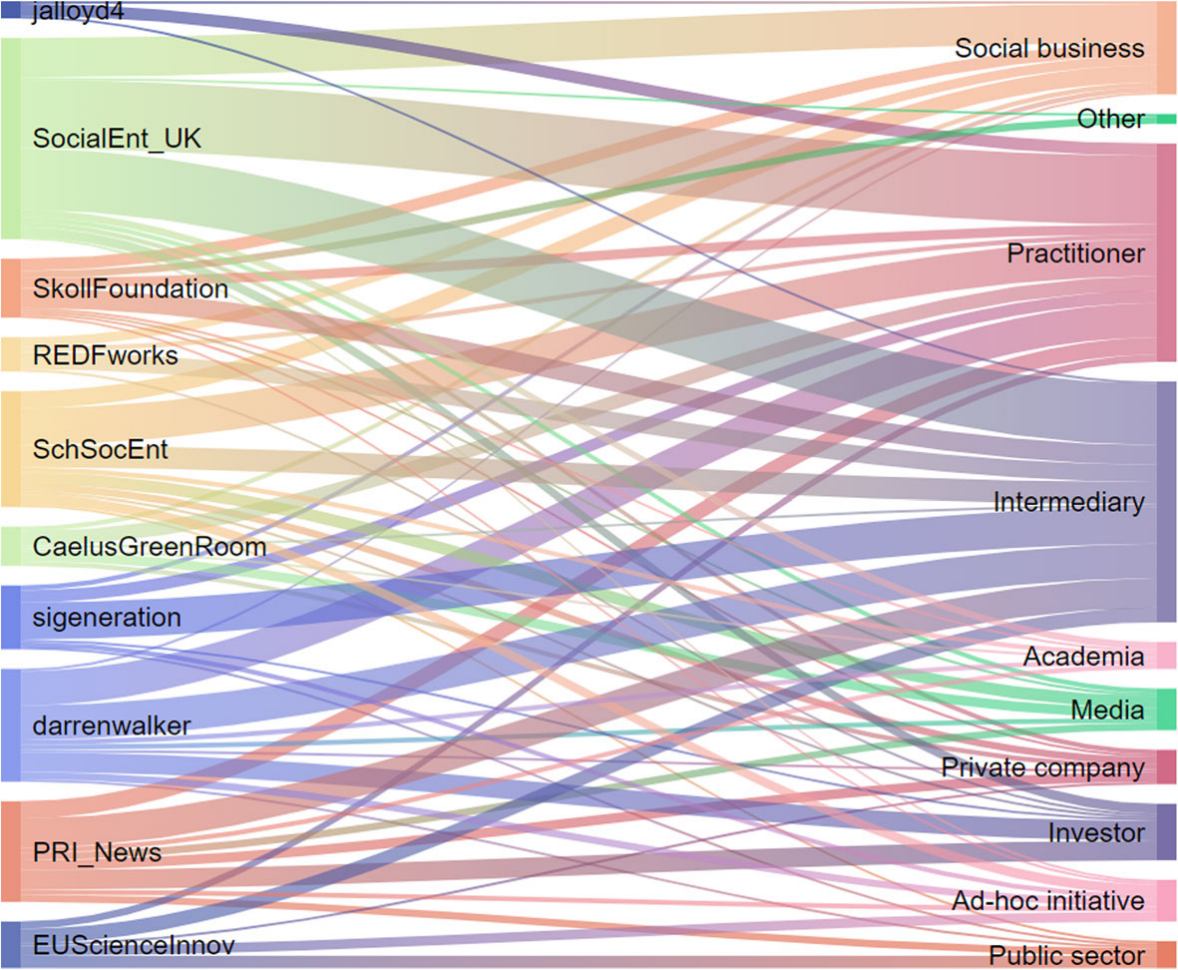
Mapping of the retweet communities (left-hand side) to influential user categories (right-hand side). Most of the retweet communities (left-hand side) have users from most of the categories (right-hand side), except the community lead by @jalloyd4, whose users are mainly practitioners with a small presence of social businesses and intermediaries

Figure 7 presents a many-to-many mapping between the retweet communities (Fig. 3) to the user categories (Table 2). Most of the retweet communities (left-hand side) have users from most of the categories (right-hand side), except the community lead by @jalloyd4, whose users are mainly practitioners with a small presence of social businesses and intermediaries. The community lead by @EUScienceInnov, the official account of the Directorate General in charge of Research and Innovation within the European Commission, has links with five user categories but surprisingly these do not include neither academia nor social business. Yet, some academics and social business representatives may be included in the “ad-hoc initiative” category, which encompasses several EU-funded project. The retweet community around @CaelusGreenRoom, a website with all the latest news on sustainability and CSR, shows most of the users are practitioners or media, but the community does not include other categories such as investors or public sector. On the other end, “Practitioners” and “Intermediaries” are spread across all the retweet communities. “Public sector” is the least “spread” (after “Other”) present in only five out of ten communities.

## Discussion

In this study, we use Twitter as a proxy to identify and understand the dynamics of the players operating within the impact investing sector. A few key points emerged from the analysis above:

**Neutral or positive sentiment towards impact investing**. Despite the low tweets volume (just 2,200 per day on average) evidencing low user engagement on the topic, most of the tweets are neutral or positive on the subject, showing favourable leaning towards impact investing. In this regard, the role of media and public sector discussed below becomes fundamental to take the sector to the next level.

**Public sector, media or academia are not among the top influential users**. Although understandably the public sector and academics may not use Twitter as regular communication channel, this results is surprising when looking at media. Journalists and bloggers, especially in the Anglo-Saxon world, are regularly posting on Twitter and English, the language of our Twitter queries, is one of the most used languages by the social media channel users (Statista 2013). The lack of media presence in our top influential users list is therefore unexpected (see point below on media for further comments).

**Communities are decentralised and diverse**. Despite being led by an influential user, retweet communities tend to be decentralised, meaning that everybody retweets everybody and not only the influencers, except in one case (Fig. 3). Furthermore, re-tweet communities include users from different sectors (Fig. 7).

**Different communities tend to use different languages**. Although a basic common language is used across all user categories and communities (i.e. #impinv and #socent are the hashtag most widely used), each user category and community tend to use a wide variety of hashtags, which may be still related to the same or similar concepts (i.e. see Fig. 5 for #socent, #socents, #socialentrepreneur, #socialentreprise) or very different themes ranging from #millenials to #education and going through #supplychain, #climatechange and #tech (Fig. 7).

Furthermore, a few broader reflections that emerged by analyzing our current dataset are highlighted in the following paragraphs.

**Hybrid organisations**. We experienced difficulties in categorising some of the users in our sample. This is mainly due to the hybrid nature of most of the organisations operating in the sector. For examples, some intermediaries are also investors (e.g., Access or Power to change), combining strong capacity building programmes with pure investment activities. Some social businesses (i.e., charities or social enterprises such as the School for Social Entrepreneurs) exhibit the same “double nature”: a strong educational purpose, offering high-level executive courses for those interested in discovering or further exploring the sector, whilst having their own status of social enterprise or charity. Although this dualism is intrinsic in the nature of the sector, there is an increasing trend of hybridisation, entailing new organisational models or cross-sectoral solutions such as public-private partnerships.

**Global phenomenon**. Despite having collected tweets in English only, the sector is impressively well spread across the world. The vast majority of tweets come from the UK and the US but our sample includes tweets from countries and cities in all continents: from Australia to Ghana, from Costa Rica to New Zealand, from Singapore to Dehli, from Milan to Dubai. This points out the global nature of the impact investing sector, which is still niche but with great potential to spread widely.

**Technological companies**. Among the 520 most influential users, 19 are companies with a focus on technology. In the previous paper (Kralj Novak et al. 2017) we noticed that this was an emerging trend across influential users. Here, with the expanded dataset and refined user categorisation, we discover that the number of tech-related users is increasing. They are mainly social businesses and private companies, indicating that some of them might see new technologies just as a tool to reach their social purposes, whilst others might have either identified the sector as their main target segment or simply are not yet aware of being a social business and therefore do not consider themselves as such. Therefore, it is worth to note that technology is playing an increasing role even in the impact investing sector, which is strongly connected to the charity world often accused of being “too traditional” or “old fashioned”. Policy makers, from national governments to international institutions, are giving increasing attention to the relationship between technology, impact investing and social innovation trends and initiatives. An example is the ICT-enabled social innovations (IESI) project launched by the Joint Research Center of the European Commission. National governments and international institutions are encouraged to do even more in this field. Social and technological progress should go ahead together if we want to live and work in sustainable societies.

**Financial investors**. Some specific categories analysed in the paper have also attracted our attention. For example, although the number of investors in the sample is not very high, there is significant diversity within the dataset: from philanthropic investors to angel, early-stage and equity investors. What is even more positively surprising is that even mainstream investment banks (such as Goldman Sachs or BNP) operate in this field. This should be interpreted as a positive sign, in order to make impact investment more mainstream. This would be a crucial step to finally drop the word “impact” before investment, simply reflecting the cultural shift in the financial sector making the so-called impact investing the new standard practice.

**Media**. This is another category included in the insights our initial research (Kralj Novak et al. 2017). The influential users in the category were just 10 out of 170 accounts (6%), and the figure has remained the same, despite the extended sample. This confirms our previous concern of a missed opportunity for media, especially in light of the positive general attitude towards the topic as demonstrated by the sentiment analysis. We note that many widely read and highly regarded magazines such as The Economist, which has a Twitter account with 23 million followers and regularly publishes articles on the issue (i.e., the last article was published on the 17th of February 2018), are still not part of our list of influencers. Similarly, we see that some others, such as Bloomberg Brief, Forbes or BBC are taking the challenge to the next level. Nonetheless, the vast majority of the most influential users are still very specialised, hence already appealing only to a speacialised audience. While this does not help policy makers in their mission of spreading the concept and reaching new audiences, the fact is that media and journalists in other countries besides the UK and US are contributing to this mission (in our sample there are users from Italy, Dubai, India, Canada, and Australia). Furthermore, policy makers and investors should take the lead and incentivise journalists and media to be more vocal in spreading the impact investing approach.

With regards to **policy makers**, the UK government is missing from our list of the most influential users posting about impact investing. This is atypical since the UK has always been regarded as one of the global leaders in the sector. Conversely, the presence of the EU-related accounts on Twitter is highly notable. For years the EU has been accused of not showing and communicating enough the results achieved by significant flows of funding going into a wide variety of development initiatives. Equally, one of the main critics expressed by the EU skeptics is the lack of transparency of the EU institutions. Of course our findings do not prove the transparency of the EU bodies, however they bring evidence of an improvement made by Brussels in sharing information and trying to engage EU citizens more widely. Furthermore, our analysis shows how active the EU is in impact investing, funding and supporting a number of initiatives in this sector. We believe that more needs to be done in this direction, not only at the EU level but also at the national and local level, with an increasing role played by local city councils, arguably the closest institution to citizens locally.

Further studies should be conducted both in English and other languages in order to capture the local nature of impact investing, as the concept is strictly interlinked with social innovation and social entrepreneurship. Equally this would allow to also understand how (and if) media frame and talk about the sector locally. We also plan to replicate the analysis in time next year, in order to be able to compare the results, map changes and capture new trends.

## Conclusions

The goal of impact investment is to generate social and environmental impact alongside a financial return. In this paper, we analyze ten months of Twitter data related to the impact investing sector and identify the main influencers and how they interact and communicate with each other. We apply network, contents and sentiment analysis on the acquired dataset. As a methodological novelty, we use the Herfindahl-Hirschman index (*HHI*) with the adapted Hirsch index (*h-index*) to study the structure of the communities. The main weakness of the current approach is limitation to English-only tweets and the use of a domain-independent sentiment classifier. Instead of the sentiment, one should better estimate the attitude (stance) towards impact investing issues by the various actors. This, however, requires considerable resources in terms of annotations by domain experts, and regular monitoring of the quality by measuring the self- and inter-annotator agreement (Mozetič et al. 2016).

The main contribution of this paper is the domain insight: our study shows that Twitter users exhibit favourable leaning (predominantly neutral or positive) towards impact investing. Communities are decentralized and include users from a variety of sectors. Despite some basic common vocabulary across retweet communities, most of the topics discussed and vocabulary used vary largely between communities. We note that an additional effort should be made in raising awareness about the sector, especially by policymakers and media outlets. The role of investors and the academia is also discussed, as well as the emergence of hybrid business models within the sector and its connections to the tech industry.
